# LINC00662 promotes cell viability and metastasis in esophageal squamous cell carcinoma by sponging miR‐340‐5p and upregulating HOXB2

**DOI:** 10.1111/1759-7714.13551

**Published:** 2020-07-07

**Authors:** Zhimei Zhang, Xuyang Liang, Ling Ren, Shuxian Zhang, Shouying Li, Tongxun Wan, Dazhou Xu, Shengxiang Lv

**Affiliations:** ^1^ Phase I Clinical Research Center The Affiliated Hospital of Kangda College of Nanjing Medical University/The First People's Hospital of Lianyungang Lianyungang Jiangsu China; ^2^ Department of Gastroenterology Lianyungang Clinical College of Nanjing Medical University /The First People’s Hospital of Lianyungang Lianyungang Jiangsu China; ^3^ Department of Gastroenterology The Affiliated Lianyungang Hospital of Xuzhou Medical University/The First People's Hospital of Lianyungang Lianyungang Jiangsu China

**Keywords:** Esophageal squamous cell carcinoma, HOXB2, LINC00662, miR‐340‐5p

## Abstract

**Background:**

Previous studies have shown that lncRNA LINC00662 plays an important role in pathogenesis of malignancies. The purpose of this study was to elucidate the regulatory mechanism of LINC00662 in esophageal squamous cell carcinoma (ESCC).

**Methods:**

In this study, the regulatory mechanism of LINC00662 was investigated by RT‐qPCR. MTT, transwell and dual luciferase reporter assays.

**Results:**

Upregulation of LINC00662 was found in ESCC and associated with worse clinical outcomes in ESCC patients. More importantly, knockdown of LINC00662 restrained cell proliferation, migration and invasion in ESCC. In addition, LINC00662 acts as a molecular sponge for miR‐340‐5p in ESCC, and miR‐340‐5p directly targets HOXB2. HOXB2 expression can be positively regulated by LINC00662 in ESCC. Furthermore, HOXB2 downregulation or miR‐340‐5p overexpression weakened the carcinogenesis of LINC00662 in ESCC.

**Conclusions:**

LncRNA LINC00662 promotes the progression of ESCC by upregulating HOXB2 by sponging miR‐340‐5p.

## Graphical abstract

1. LINC00662 functions as a novel oncogenic lncRNA in ESCC.
2. LINC00662 promotes the progression of ESCC by upregulating HOXB2 by sponging miR‐340‐5p.

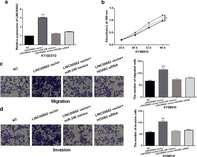

## Introduction

Esophageal squamous cell carcinoma (ESCC) is one of the most frequent malignant tumors in China and more than 90% of esophageal cancer in China is squamous cell carcinoma.^1^ Its mortality rate is second only to gastric cancer. Currently, genetic factors and dietary habits are the main causes of esophageal cancer.^2^ The main treatment for early ESCC is radical surgery. After surgery, preventive chemotherapy and radiotherapy are also needed to prevent the recurrence of the tumor. For advanced ESCC, the main treatment methods are chemotherapy, radiotherapy, immunotherapy, and targeted drug therapy.^3^ The overall five‐year survival rate of ESCC is approximately 20%, and only early superficial cancer can be cured. The survival rate of patients with ESCC is mainly related to the TNM stage, tumor morphology and molecular biological characteristics.^4^ Therefore, it is very important to explore new molecular markers for early diagnosis of patients with ESCC.

Long non‐coding RNA (lncRNA) exerts its biological functions through a variety of mechanisms, including gene imprinting, chromatin remodeling, cell cycle regulation, splicing regulation, mRNA degradation and translation regulation.^5^ LncRNA can regulate gene expression through these mechanisms to regulate tumorigenesis, including ESCC. For example, silencing of lncRNA SNHG6 inhibited ESCC progression via miR‐186/HIF1α axis.^6^ LncRNA ZFAS1 promoted the proliferation, migration and invasion of ESCC cells and inhibited apoptosis via miR‐124/STAT3 axis.^7^ Recently, the role of LINC00662 in human diseases attracted our attention. Upregulation of LINC00662 has been detected in colorectal cancer and chordoma.^8^, ^9^ Functionally, LINC00662‐miR‐15b‐5p mediated GPR120 dysregulation contributed to osteoarthritis.,^10^ and lncRNA LINC00662 was found to promote colon cancer tumor growth and metastasis by competitively binding with miR‐340‐5p to regulate CLDN8 expression.^11^ miR‐340 has been reported to inhibit ESCC cell growth and invasion by targeting phosphoserine aminotransferase 1.^12^ However, the regulatory mechanism of LINC00662/miR‐340‐5p is still unclear in ESCC.

In this study, Homeobox B2 (HOXB2) was predicted to be a potential target for miR‐340‐5p. HOXB2 has been shown to participate in pathogenesis of human cancers. It has been reported that HOXB2 was upregulated in cervical cancer and pancreatic cancer.^13^, ^14^ In addition, miR‐4324 has been found to function as a tumor suppressor in colorectal cancer by targeting HOXB2,^15^ and lncRNA HOXB‐AS1 promoted proliferation, migration and invasion of glioblastoma cells via miR‐885‐3p/HOXB2 axis.^16^ Moreover, HOXB2 has been demonstrated to be a target of miR‐340 in acute myeloid leukemia.^17^ However, the role of HOXB2 in ESCC has not been reported in previous studies.

Therefore, the function of HOXB2 as well as LINC00662/miR‐340‐5p was investigated in ESCC. More importantly, the regulatory mechanism of LINC00662/miR‐340‐5p/HOXB2 in ESCC was elucidated in this study. This research may provide a novel hallmark for early diagnosis and treatment of ESCC.

## Methods

### Clinical tissues

A total of 72 surgical tumor specimens and adjacent tissue samples were obtained from The Affiliated Lianyungang Hospital of Xuzhou Medical University/The First People's Hospital of Lianyungang. All patients with ESCC provided their written informed consents and received no treatment prior to surgery. Human tissues were frozen in liquid nitrogen and then stored in a −80°C refrigerator for further experiments. This study was approved by the Institutional Ethics Committee of The Affiliated Lianyungang Hospital of Xuzhou Medical University/The First People's Hospital of Lianyungang.

### Cell cultures and cell transfection

Normal esophageal cell line (Het‐1A) and ESCC cell lines (KYSE140, KYSE510) were purchased from the Institute of Biochemistry and Cell Biology, Chinese Academy of Sciences (Shanghai, China). All cells were seeded in RPMI‐1640 medium (Gibco, Carlsbad, CA, USA) containing 10% fetal bovine serum (FBS) and cultured at 37°C with 5% CO_2_.

LINC00662 siRNA and vector, miR‐340‐5p mimics and inhibitor, HOXB2 siRNA were purchased from GenePharma (Shanghai, China). They were transferred into KYSE510 cells using Lipofectamine 2000 (Invitrogen, Carlsbad, CA, USA).

### Quantitative RT‐PCR

TRIzol reagent (Invitrogen, USA) was applied for extracting total RNA in ESCC tissues and cell lines. 1000 μg of total RNA and Prime‐Scrip One Step RT‐PCR Kit (TaKaRa, Dalian, China) were used for the synthesis of cDNA. Quantitative RT‐PCR was conducted using the SYBR Green Master Mix (Roche, USA) on ABI7500 thermocycler (Thermo Fisher Scientific, Inc.). U6 and GAPDH were used as control for LINC00662, miR‐340‐5p and HOXB2. And their expressions were calculated using the 2^−△△ct^ method.

### Luciferase reporter assay

The 3′‐UTR of wild‐type or mutant LINC00662 and HOXB2 was inserted into the pGL3 promoter vector (Invitrogen, USA). Then, the pGL3 promoter vector and miR‐340‐5p mimics were transfected into KYSE510 cells. Finally, the dual luciferase reporter system (Promega, USA) was applied to measure luciferase activities.

### Western blot analysis

Protein samples were obtained using RIPA lysis buffer. The protein content was calculated using bicinchoninic acid (BCA). Then, an equal amount of proteins (30 μg) was separated by 10% SDS‐PAGE and transferred to polyvinylidene difluoride (PVDF) membranes (Thermo Fisher Scientific, Inc.). The membranes were blocked with 5% skim milk at room temperature for one hour. Next, the protein samples were incubated with HOXB2 and GAPDH primary antibodies (Abcam, Cambridge, MA, USA) at 4°C overnight. After washing, they were incubated with goat polyclonal anti‐rabbit IgG secondary antibody (Abcam, USA) for two hours at room temperature. Finally, the protein was measured by ECL (ECL, Pierce).

### MTT assay

The MTT (3‐(4, 5‐dimethyl‐2‐thiazolyl)‐2, 5‐diphenyl‐2H‐tetrazolium bromide) assay was applied to measure cell proliferation. Transfected KYSE510 cells (1 × 10^3^/well) were seeded onto 96‐well plates and incubated for 0–96 hours. Next, MTT (Sigma, MO) was added to the cells and they were incubated for four hours at 37°C. The absorbance at 490 nm (OD = 490 nm) was detected using a spectrophotometer.

### Transwell assay

Cell migration and invasion were assessed by Transwell assay. Transwell chambers were coated with Matrigel (BD Biosciences, NJ, USA) to detect cell invasion, and 5 × 10^3^ transfected KYSE510 cells were seeded into the upper chambers (8 μm pore size; Corning Incorporated, Corning, NY, USA). RPMI‐1640 medium with 10% FBS was then placed into the lower chamber. The cells were incubated at 37°C with 5% CO_2_ for 24 hours. The invasive cells on the lower surface were fixed with 4% PFA and stained with 0.1% crystal violet. Cell migration was detected without Matrigel and the migrated cells were counted under a light microscope.

### Statistical analysis

Data are shown as mean ± SD. Statistical analysis was performed using GraphPad Prism 6.0 and SPSS 19.0. Difference was calculated by Student *t*‐test or one‐way ANOVA. *P* < 0.05 was considered statistically significant.

## Results

### LINC00662 enhances malignancy of ESCC

**Figure 1:**
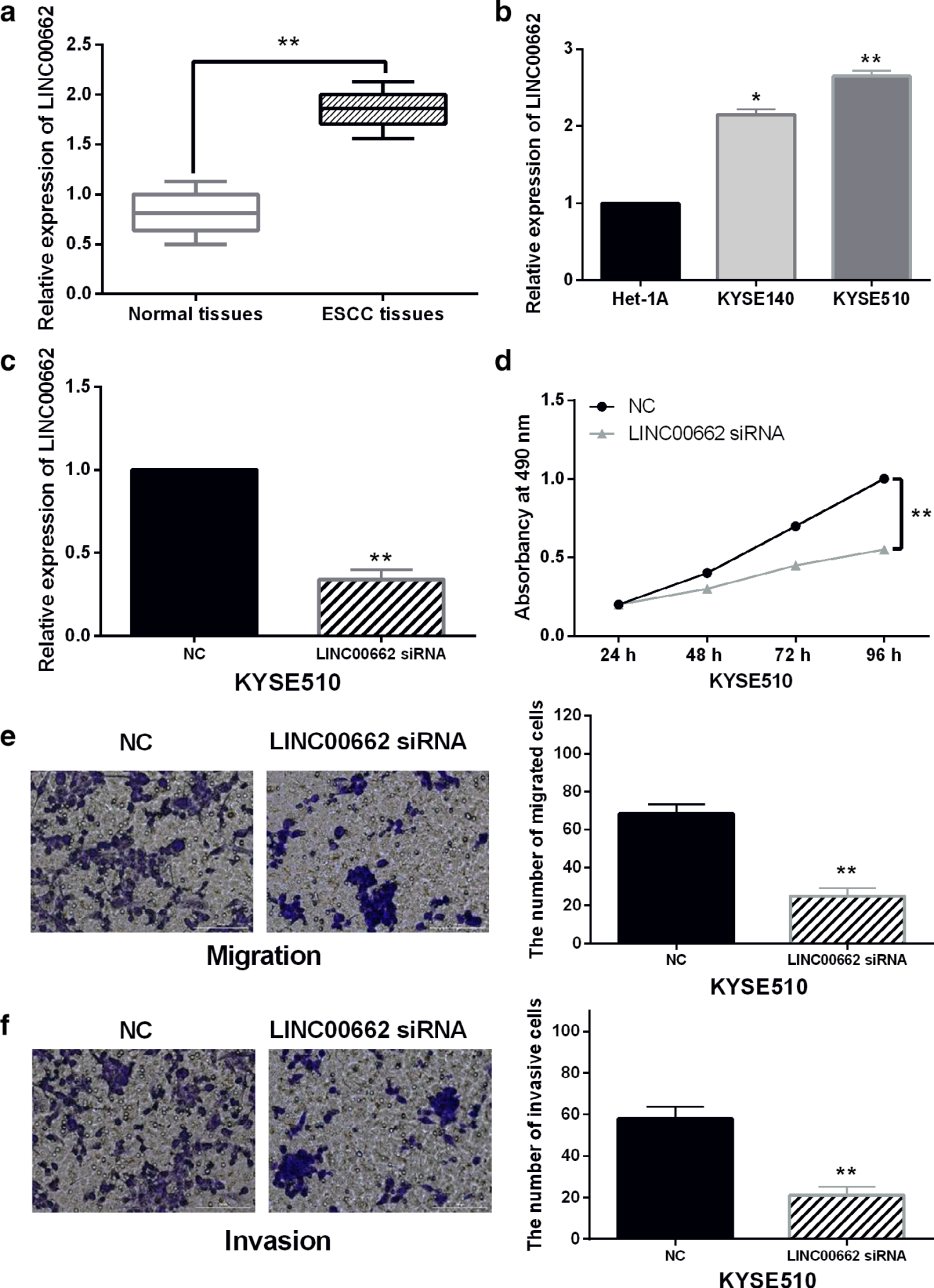
LINC00662 enhances malignancy of ESCC. (**a**, **b**) The expression of LINC00662 in ESCC tissues and cells (**c**) LINC00662 expression in KYSE510 cells with LINC00662 siRNA. (**d**, **e**, **f**) Cell proliferation, migration and invasion in KYSE510 cells with LINC00662 siRNA *P < 0.05, **P < 0.01. (**d**) (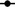) NC, (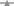) LINC00662 siRNA

First, the expression level of LINC00662 was examined in ESCC tissues by RT‐qPCR. LINC00662 expression in ESCC was higher than that in normal tissues (Fig 1a). We found that the abnormal expression of LINC00662 was associated with differentiation, TNM stage and lymph‐node metastasis in ESCC patients (*P* < 0.05, Table 1). Compared to normal esophageal cell line Het‐1A, upregulation of LINC00662 was detected in ESCC cell lines KYSE140 and KYSE510 (Fig 1b). Due to the significant difference of LINC00662 expression in KYSE510 cells, KYSE510 cells were used for further experiments. To explore the function of LINC00662 in ESCC, LINC00662 siRNA was transfected into KYSE510 cells. RT‐qPCR showed that LINC00662 siRNA decreased its expression in KYSE510 cells (Fig 1c). MTT assay suggested that downregulation of LINC00662 suppressed the proliferation of KYSE510 cells (Fig 1d). Transwell assay indicated that LINC00662 downregulation inhibited cell migration and invasion in KYSE510 cells (Fig 1e,f). Collectively, LINC00662 serves as an oncogene in the progression of ESCC.

**Table 1:** Relationship between LINC00662 expression and the clinicopathological characteristics in 72 ESCC patients

	Number of cases (*n* = 72)	LINC00662		
Characteristics		High	Low	*P*‐value
**Age (years)**				0.26
≥60	42	30	12	
<60	30	20	10	
**Gender**				0.51
Male	39	29	10	
Female	33	21	12	
**Tumor size (cm)**				0.11
≥4	29	21	8	
<4	43	29	14	
**Differentiation**				0.03*
Well and moderately	40	33	7	
Poorly	32	17	15	
**TNM stage**				0.02*
I + II	46	33	13	
III + IV	26	17	9	
**Lymph‐node metastasis**				
Negative	48	32	16	0.02*
Positive	24	18	6	

* *P*<0.05 was considered significant.

Statistical analyses were performed by the χ2 test.

TNM, tumor‐node‐metastasis.

### LINC00662 directly interacts with miR‐340‐5p in ESCC

**Figure 2:**
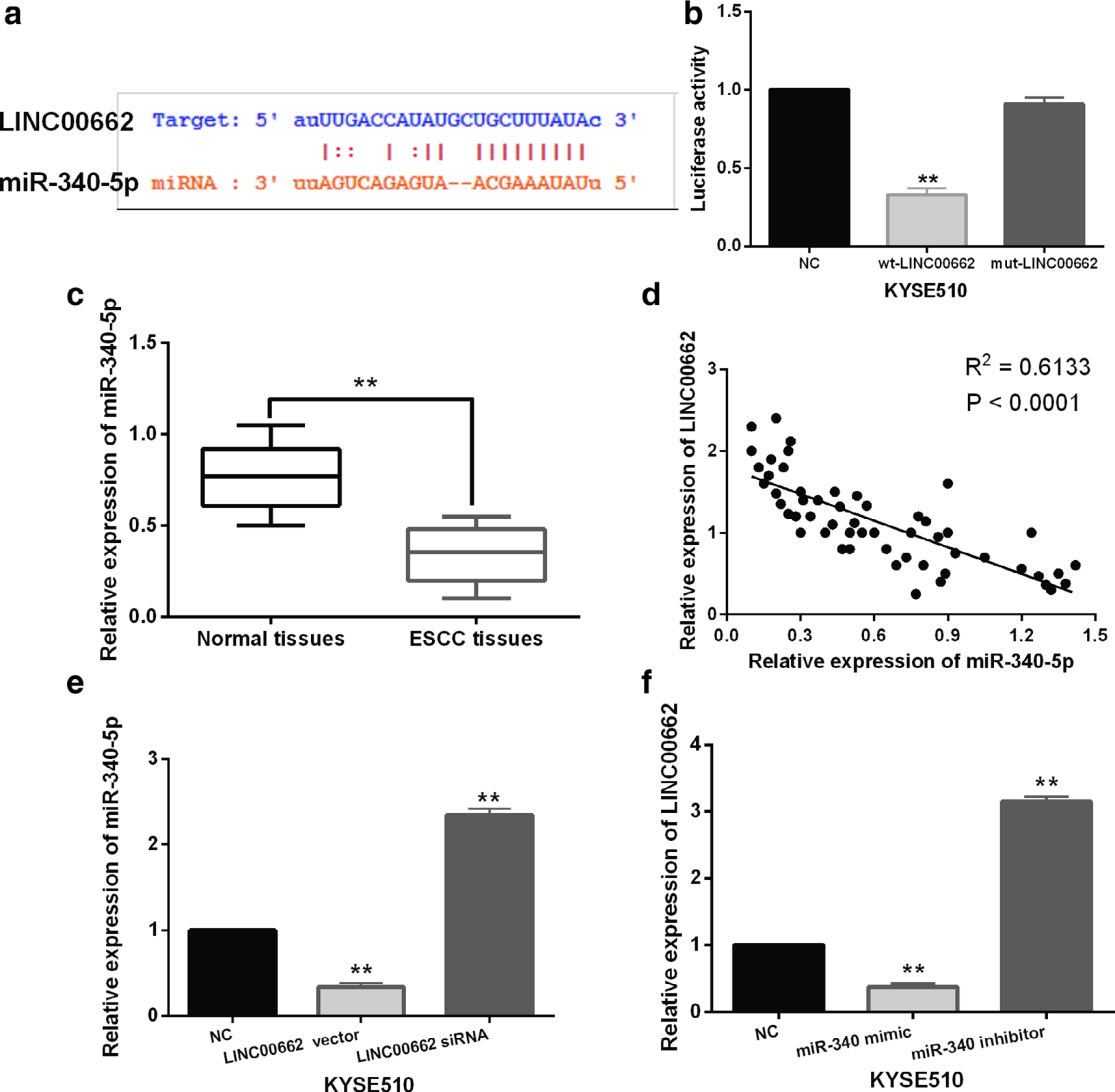
LINC00662 directly interacts with miR‐340‐5p in ESCC. (**a**) The binding sites between LINC00662 with miR‐340‐5p. (**b**) Luciferase reporter assay (**c**) MiR‐340‐5p expression in ESCC tissues and normal tissues (**d**) A negative correlation between LINC00662 and miR‐340‐5p expression in ESCC tissues (**e**) MiR‐340‐5p expression in KYSE510 cells with LINC00662 siRNA or vector (**f**) LINC00662 expression in KYSE510 cells containing miR‐340‐5p mimics or inhibitor **P < 0.01.

The starBase version 2.0 (http://starbase.sysu.edu.cn/) predicts that LINC00662 has a binding site with miR‐340‐5p (Fig 2a). To verify the above prediction, dual luciferase reporter was designed. We found that miR‐340‐5p mimics reduced the luciferase activity of wt‐LINC00662 in KYSE510 cells (Fig 2b), indicating that LINC00662 can directly bind to miR‐340‐5p in ESCC. In addition, miR‐340‐5p expression was detected in ESCC tissues. Compared to normal tissues, miR‐340‐5p expression was decreased in ESCC tissues (Fig 2c), and the abnormal expression of miR‐340‐5p was related to differentiation, TNM stage and lymph node metastasis in ESCC patients (*P* < 0.05, Table 2). We then found that LINC00662 was negatively correlated with miR‐340‐5p expression in ESCC tissues (Fig 2d). Consistently, miR‐340‐5p expression was decreased by LINC00662 upregulation and increased by LINC00662 downregulation in KYSE510 cells (Fig 2e). Meanwhile, miR‐340‐5p overexpression reduced LINC00662 expression, while miR‐340‐5p downregulation promoted LINC00662 expression in KYSE510 cells (Fig 2f). Therefore, LINC00662 acts as a molecular sponge for miR‐340‐5p in ESCC.

**Table 2:** Relationship between the expression of miR‐340‐5p or HOXB2 and clinicopathological characteristics in 72 ESCC patients

		miR‐340‐5p			HOXB2		
Characteristics	Cases	High	Low	*P*‐value	High	Low	*P*‐value
**Age (years)**				0.26			0.31
≥60	42	12	30		25	17	
<60	30	9	21		20	10	
**Gender**				0.33			0.41
Male	39	13	26		27	12	
Female	33	8	25		18	15	
**Tumor size (cm)**				0.08			0.04*
≥4	29	10	19		20	9	
<4	43	11	32		25	18	
**Differentiation**				0.04*			0.06
Well and moderately	40	14	26		26	14	
Poorly	32	7	25		19	13	
**TNM stage**				0.03*			0.04*
I + II	46	15	31		25	21	
III + IV	26	6	20		20	6	
**Lymph‐node metastasis**				0.02*			0.03*
Negative	48	16	32		30	18	
Positive	24	5	19		15	9	

* *P*<0.05 was considered significant.

Statistical analyses were performed using the χ2 test.

### HOXB2 is a direct target of miR‐340‐5p

**Figure 3:**
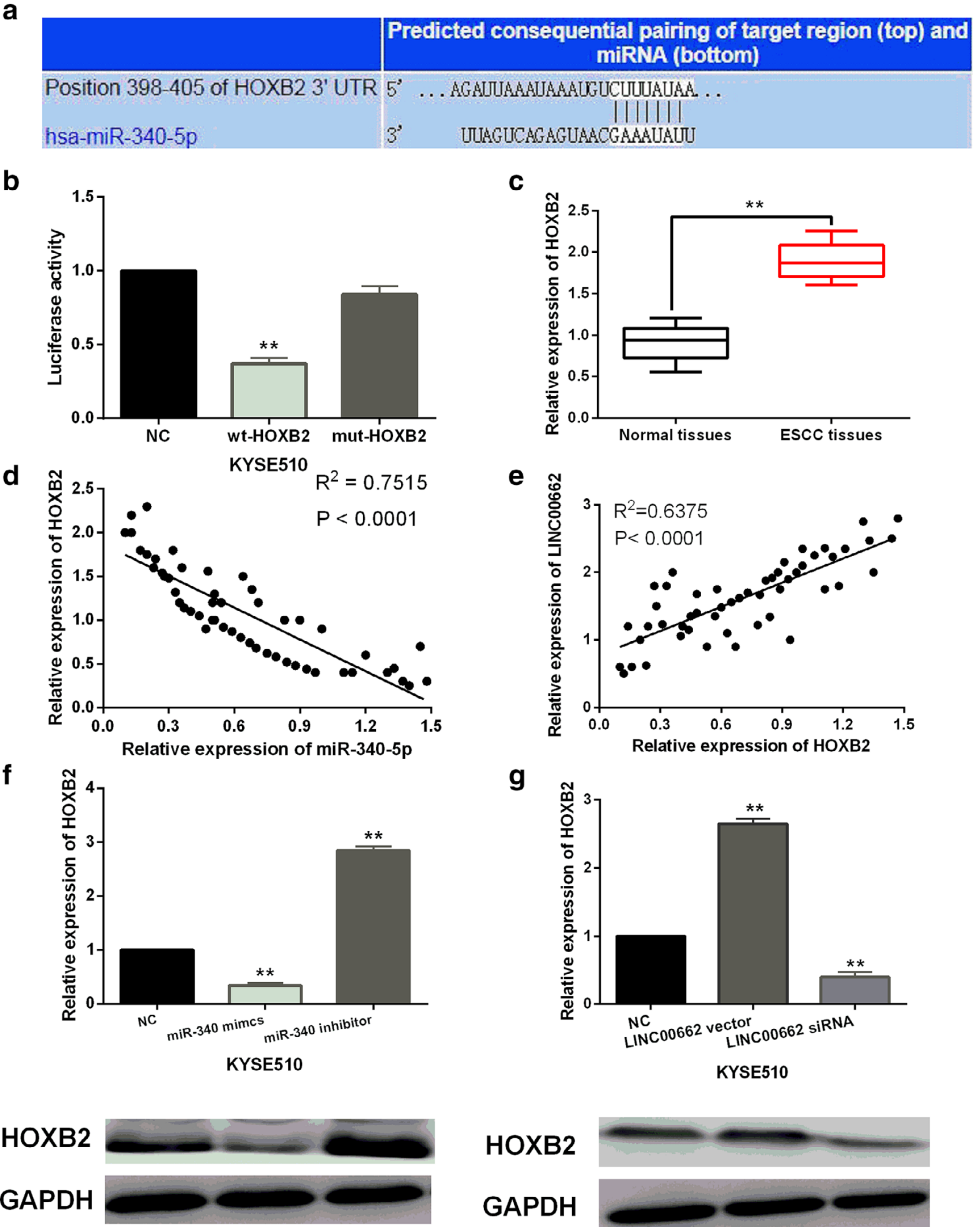
HOXB2 is a direct target of miR‐340‐5p. (**a**) The binding sites between miR‐340‐5p and HOXB2. (**b**) Luciferase reporter assay (**c**) HOXB2 expression in ESCC tissues and normal tissues (**d**) A negative correlation between miR‐340‐5p and HOXB2 expression was detected in ESCC tissues (**e**) A positive correlation between LINC00662 and HOXB2 expression was found in ESCC tissues (**f**) HOXB2 mRNA and protein expression in KYSE510 cells with miR‐340‐5p mimics or inhibitor (**g**) HOXB2 mRNA and protein expression in KYSE510 cells with LINC00662 siRNA or vector ** *P* < 0.01.

Further, TargetScan database (http://www.targetscan.org) shows that miR‐340‐5p has a binding site with the 3’‐UTR of HOXB2 (Fig 3a). Dual luciferase reporter assay showed that miR‐340‐5p mimics reduced the luciferase activity of wt‐HOXB2, but had little effect on that of mut‐HOXB2 in KYSE510 cells (Fig 3b). Next, the expression level of HOXB2 was examined in ESCC tissues. HOXB2 was found to be upregulated in ESCC tissues compared to normal tissues (Fig 3c). Moreover, the abnormal expression of HOXB2 was related to tumor size, TNM stage and lymph node metastasis in ESCC patients (*P* < 0.05, Table 2). A negative correlation between miR‐340‐5p and HOXB2 expression was found in ESCC tissues (Fig 3d). Meanwhile, LINC00662 was found to be positively correlated with HOXB2 expression in ESCC tissues (Fig 3e). In addition, we found that the mRNA and protein expression of HOXB2 was reduced by miR‐340‐5p overexpression and promoted by miR‐340‐5p downregulation in KYSE510 cells (Fig 3f), and LINC00662 upregulation enhanced HOXB2 mRNA and protein expression, while LINC00662 downregulation declined HOXB2 mRNA and protein expression in KYSE510 cells (Fig 3g). Taken together, miR‐340‐5p directly targets HOXB2 in ESCC, and HOXB2 expression can be positively regulated by LINC00662 in ESCC.

### HOXB2 regulates ESCC progression by participating in LINC00662/miR‐340‐5p axis

**Figure 4:**
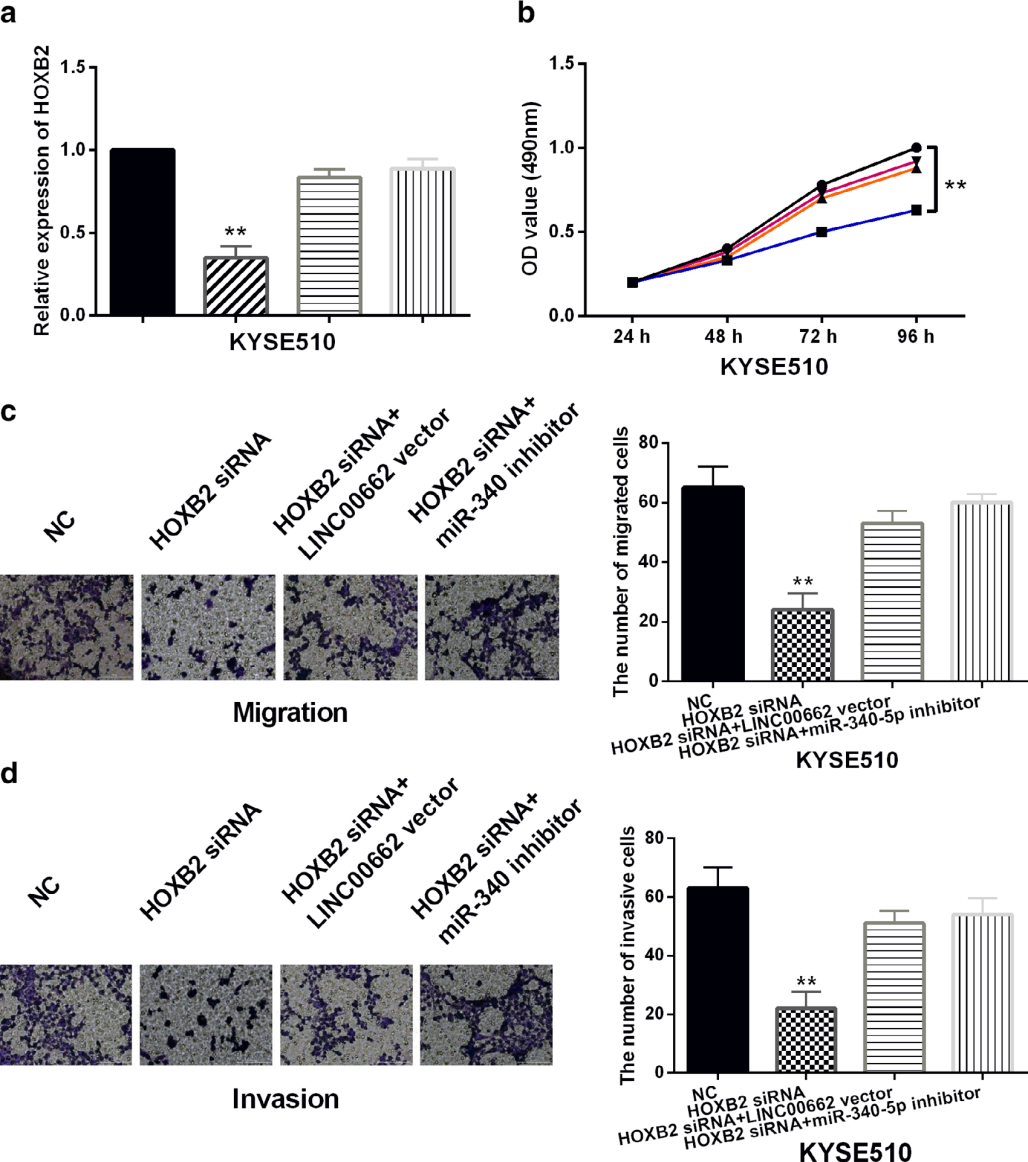
HOXB2 regulates ESCC progression by participating in LINC00662/miR‐340‐5p axis. (**a**) HOXB2 expression in KYSE510 cells with HOXB2 siRNA, HOXB2 siRNA+LINC00662 vector or HOXB2 siRNA+miR‐340‐5p inhibitor (**b**,**c**,**d**) Cell proliferation, migration and invasion in KYSE510 cells with HOXB2 siRNA, HOXB2 siRNA+LINC00662 vector or HOXB2 siRNA+miR‐340‐5p inhibitor. **P < 0.01. (**a**) (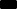) NC, (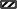) HOXB2 siRNA, (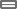) HOXB2 siRNA+LINC00662 vector, (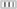) HOXB2 siRNA+miR‐340‐5p inhibitor; (**b**) (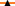) HOXB2 siRNA+LINC00662 vector, (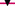) HOXB2 siRNA+miR‐340‐5p inhibitor, (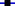) HOXB2 siRNA, (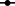) NC.

Next, HOXB2 siRNA, HOXB2 siRNA+LINC00662 vector or HOXB2 siRNA+miR‐340‐5p inhibitor were cotransfected into KYSE510 cells. HOXB2 expression was reduced by its siRNA in KYSE510 cells. LINC00662 vector and miR‐340‐5p inhibitor restored the reduction of HOXB2 expression induced by HOXB2 siRNA (Fig 4a). In addition, knockdown of HOXB2 inhibited KYSE510 cell proliferation, and LINC00662 upregulation or miR‐340‐5p downregulation weakened the inhibitory effect of HOXB2 siRNA on cell proliferation (Fig 4b). Transwell assay showed that HOXB2 downregulation suppressed cell migration and invasion in KYSE510 cells. The inhibitory effect of HOXB2 siRNA on KYSE510 cell migration and invasion was also abolished by LINC00662 vector and miR‐340‐5p inhibitor (Fig 4c,d). Collectively, HOXB2 serves as an oncogene in ESCC.

### LINC00662 promotes ESCC progression by regulating miR‐340‐5p/HOXB2 axis

**Figure 5:**
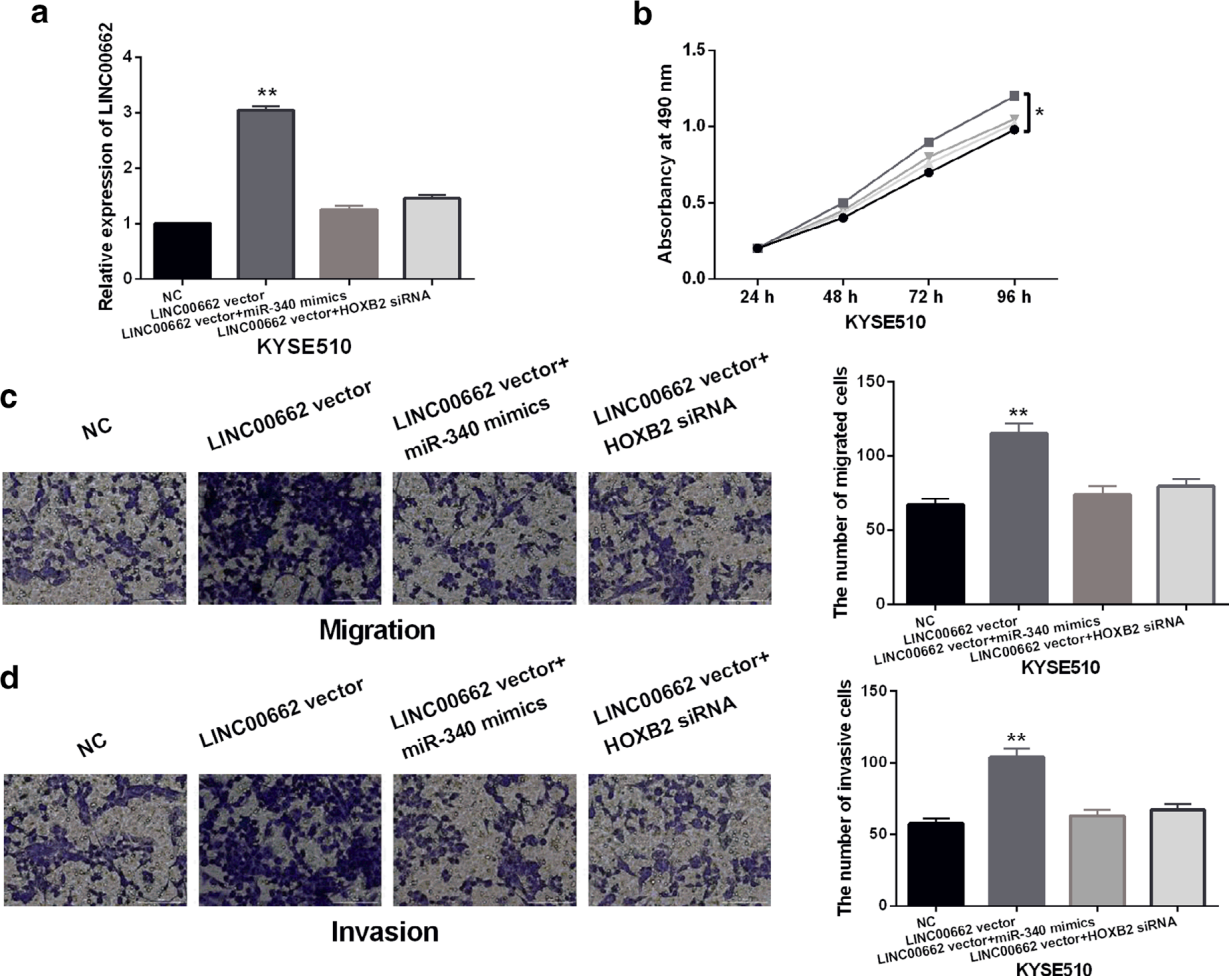
LINC00662 promotes ESCC progression by regulating miR‐340‐5p/HOXB2 axis. (**a**) LINC00662 expression in KYSE510 cells with LINC00662 vector, HOXB2 siRNA+LINC00662 vector or LINC00662 vector+miR‐340‐5p mimics. (**b**, **c**, **d**) Cell proliferation, migration and invasion in KYSE510 cells with LINC00662 vector, HOXB2 siRNA+LINC00662 vector or LINC00662 vector+miR‐340‐5p mimics *P < 0.05, **P < 0.01. (**b**) (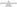) LINC00662 vector+miR‐340‐5p mimics, (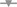) LINC00662 vector+HOXB2 siRNA, (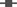) LINC00662 vector, (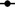) NC.

To explore the interaction between LINC00662 and miR‐340‐5p or HOXB2 in ESCC, HOXB2 siRNA or miR‐340‐5p mimics was transfected into KYSE510 cells with LINC00662 vector. RT‐qPCR showed that the increased expression of LINC00662 induced by its vector was abolished by HOXB2 siRNA or miR‐340‐5p mimics in KYSE510 cells (Fig 5a). Functionally, LINC00662 upregulation was found to promote cell proliferation, migration and invasion in KYSE510 cells. More importantly, HOXB2 downregulation or miR‐340‐5p overexpression weakened the promoting effect of LINC00662 on cell proliferation, migration and invasion in KYSE510 cells (Fig 5b–d). These results indicate that LINC00662 promotes the progression of ESCC by regulating miR‐340‐5p/HOXB2 axis.

## Discussion

In recent years, many lncRNAs have been shown to play important roles in the process of tumor formation at both the transcription and post‐transcription levels. Previous studies have demonstrated that abnormal expression of lncRNAs can regulate tumor growth, metastasis and apoptosis in human cancers by acting as tumor promoter or inhibitor. Meanwhile, various lncRNAs have reported to play vital roles in progression of ESCC. For example, lncRNA SNHG12 was downregulated and suppressed tumor progression in ESCC.^18^ In addition, upregulation of lncRNA DLX6‐AS1 was detected in ESCC and promoted ESCC cell growth and metastasis via targeting miR‐577.^19^ The functions of LINC00662 were also investigated in human cancers. Upregulation of LINC00662 has been found in hepatocellular carcinoma and acute myeloid leukemia.^20^, ^21^ Functionally, LINC00662 functioned as an miRNA sponge to promote the prostate cancer tumorigenesis through targeting miR‐34a,^22^ and LINC00662 was also found to promote proliferation and migration in oral squamous cell carcinoma.^23^ These results imply that LINC00662 usually acts as an oncogene in human cancers.

Consistently, LINC00662 was also upregulated in ESCC tissues and cells, and upregulation of LINC00662 was associated with worse clinical outcomes in ESCC patients. Functionally, upregulation of LINC00662 promoted cell proliferation, migration and invasion in ESCC. These results indicate that LINC00662 also serves as a tumor promoter that stimulates malignant progression of OSCC. This is the first report to evaluate the function of LINC00662 in human ESCC. However, a limitation in this study is that the effect of LINC00662 on cell cycle which is the key phenomenon in cell survival has not been confirmed in ESCC. Further, LINC00662 acts as a molecular sponge for miR‐340‐5p in ESCC. Overexpression of miR‐340‐5p weakened the carcinogenesis of LINC00662 in ESCC, indicating that miR‐340‐5p play an antitumor effect in ESCC.

Similar to our results, Yan *et al*. found that miR‐340 was downregulated in ESCC, and overexpression of miR‐340 inhibited ESCC cell viability, invasion and EMT.^12^ In addition, lncRNA LINC00662 has been reported to promote colon cancer tumor growth and metastasis by competitively binding with miR‐340‐5p.^11^ In this study, we also found that LINC00662 promoted cell viability and metastasis in ESCC by sponging miR‐340‐5p. In addition, miR‐340‐5p was confirmed to directly target HOXB2. HOXB2 expression can be positively regulated by LINC00662 in ESCC. Functionally, HOXB2 downregulation weakened the carcinogenesis of LINC00662 in ESCC. Here, upregulation of HOXB2 was examined in ESCC. Functionally, knockdown of HOXB2 inhibited cell proliferation, migration and invasion in ESCC. Consistent with our results, upregulation and carcinogenesis of HOXB2 have also been identified in osteosarcoma and ovarian cancer.^24^, ^25^ Moreover, lncRNA HOXB‐AS1 promoted proliferation, migration and invasion of glioblastoma cells via miR‐885‐3p/HOXB2 axis.^16^ It has also been reported that lncRNA RGMB‐AS1 promoted glioma growth and invasion through miR‐1200/HOXB2 axis.^26^ In the present study, LINC00662 also facilitated ESCC progression by regulating miR‐340‐5p/HOXB2 axis.

In conclusion, this study demonstrates LINC00662 as a novel oncogenic lncRNA in ESCC. A ceRNA regulatory axis of LINC00662/miR‐340‐5p/HOXB2 was identified. Specifically, LINC00662 promotes the progression of ESCC by upregulating HOXB2 by sponging miR‐340‐5p. Our findings indicate that LINC00662/miR‐340‐5p/HOXB2 axis may be potential targets for the treatment of ESCC.

## Funding

This study is supported by Medical disciplines construction project of Department of Gastroenterology (ZD201929).

## Disclosure

No authors report any conflict of interest.
